# Irisin Preserves Cardiac Performance and Insulin Sensitivity in Response to Hemorrhage

**DOI:** 10.3390/ph15101193

**Published:** 2022-09-27

**Authors:** Supaporn Kulthinee, Lijiang Wang, Naohiro Yano, Patrycja M. Dubielecka, Ling X. Zhang, Shougang Zhuang, Gangjian Qin, Yu Tina Zhao, Yue Eugene Chin, Ting C. Zhao

**Affiliations:** 1 Department of Surgery, Rhode Island Hospital, Brown University, Providence, RI 02903, USA; 2 Department of Medicine, Rhode Island Hospital, Warren Alpert Medical School of Brown University, Providence, RI, USA; 3 Department of Biomedical Engineering, University of Alabama at Birmingham, Birmingham, AL 35294, USA; 4 University of Rochester Medical Center, Rochester, NY 14642, USA; 5 Translation Medicine Center, Shanghai Chest Hospital, Shanghai Jiao Tong University, Shanghai 200240, China; 6 Department of Surgery and Plastic Surgery, Rhode Island Hospital, Brown University, Providence, RI 02903, USA

**Keywords:** irisin, hemorrhage, myocardial function, insulin resistance, inflammation

## Abstract

Irisin, a cleaved product of the fibronectin type III domain containing protein-5, is produced in the muscle tissue, which plays an important role in modulating insulin resistance. However, it remains unknown if irisin provides a protective effect against the detrimental outcomes of hemorrhage. Hemorrhages were simulated in male CD-1 mice to achieve a mean arterial blood pressure of 35–45 mmHg, followed by resuscitation. Irisin (50 ng/kg) and the vehicle (saline) were administrated at the start of resuscitation. Cardiac function was assessed by echocardiography, and hemodynamics were measured through femoral artery catheterization. A glucose tolerance test was used to evaluate insulin sensitivity. An enzyme-linked immunosorbent assay was performed to detect inflammatory factors in the muscles and blood serum. Western blot was carried out to assess the irisin production in skeletal muscles. Histological analyses were used to determine tissue damage and active-caspase 3 apoptotic signals. The hemorrhage suppressed cardiac performance, as indicated by a reduced ejection fraction and fractional shortening, which was accompanied by enhanced insulin resistance and hyperinsulinemia. Furthermore, the hemorrhage resulted in a marked decrease in irisin and an increase in the production of tumor necrosis factor-α (TNF-α) and interleukin-1 (IL-1). Additionally, the hemorrhage caused marked edema, inflammatory cell infiltration and active-caspase 3 positive signals in skeletal muscles and cardiac muscles. Irisin treatment led to a significant improvement in the cardiac function of animals exposed to a hemorrhage. In addition, irisin treatment improved insulin sensitivity, which is consistent with the suppressed inflammatory cytokine secretion elicited by hemorrhages. Furthermore, hemorrhage-induced tissue edema, inflammatory cell infiltration, and active-caspase 3 positive signaling were attenuated by irisin treatment. The results suggest that irisin protects against damage from a hemorrhage through the modulation of insulin sensitivity.

## 1. Introduction

Hemorrhagic shock can result from severe traumatic injury, causing significant complications and high mortality due to excessive blood loss [1] and, ultimately, multi-organ failure [2]. Hemorrhagic shock is a major health complication that produces serious consequences, and thus, improving our understanding of effective treatment is of paramount importance. Irisin is a recently described myokine composed of 112 amino acids that were first described by Boström and his colleagues in 2012. Irisin is produced from the proteolytic cleavage of its precursor transmembrane protein fibronectin III domain, containing protein-5 (FNDC5) [3]. The FNDC5 has been found to be highly expressed in a variety of organs, mainly produced in skeletal muscle and is considered a new hormone factor [4]. Since its discovery, irisin has been shown to increase energy expenditure in response to exercise and, because of its efficacy, has been suggested as a potential therapeutic agent for metabolic disorders in humans. Since this discovery, a multitude of evidence has suggested that irisin serves as a critical regulator in attenuating insulin resistance, promoting improved mitochondrial function, and countering inflammation [5,6,7,8]. Studies have focused on determining the crucial role that irisin plays in the pathophysiological condition of metabolic disease. Our previous study reported how irisin attenuates insulin resistance and mitigates cardiac dysfunction in high-fat, diet-induced metabolic disorders through the HDAC4 pathway [9]. In addition, it was reported that t injuries, infections, and other critical illnesses are often associated with hyperglycemia, hyperinsulinemia, and insulin resistance which contribute critically to the development of trauma and hemorrhages [10]. We showed the protective effect of irisin on myocardial ischemia/reperfusion injuries by the suppression of cell apoptosis in an acute myocardial ischemia and reperfusion model [11]. In addition, we demonstrated that irisin treatment attenuated myocardial remodeling and improved cardiac function in the cell-transplanted, infarcted heart [12]. In this study, we employed the mouse hemorrhage and resuscitation model to identify whether irisin production would be decreased in response to a hemorrhage and if the treatment of hemorrhagic mice with irisin would manifest protection against the detrimental effects that follow a hemorrhage, which is related to the regulation of insulin sensitivity. By using an established animal hemorrhage and resuscitation mouse model along with analyzing the cardiac function, insulin resistance, inflammatory factors, and tissue pathological index, we have found that irisin plays a critical role against the detrimental effects of hemorrhage as evident in the improved cardiac function, enhanced insulin sensitivity, and decreased production of inflammatory factors.

## 2. Results

### 2.1. Effects of Irisin on Hemodynamics and Cardiac Performance in Mice Exposed to Hemorrhage

**Figure 1:**
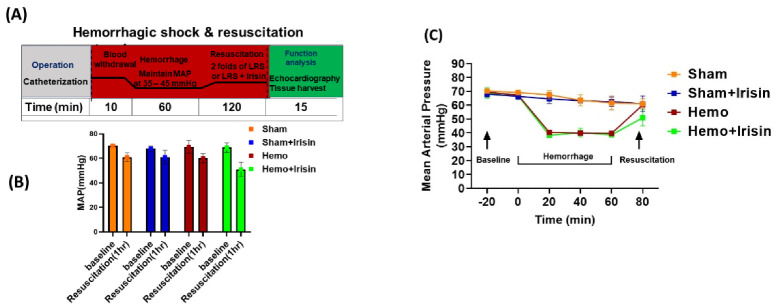
Time course and experimental protocol for the hemorrhagic shock model. (**A**) BP of mice were maintained at 35-45 mmHg of mean arterial blood pressure (MAP) for one hour before fluid resuscitation with lactated ringer’s solution (LRS) in hemorrhagic shock groups. Irisin treatment was conducted simultaneously with resuscitation. The sham groups underwent operation without blood withdrawal; (**B**) MAP of animals while in hemorrhagic shock (Data are shown as Mean ± SEM, n = 4/each group); (**C**) The time course of MAP during the hemorrhage.

**Figure 2:**
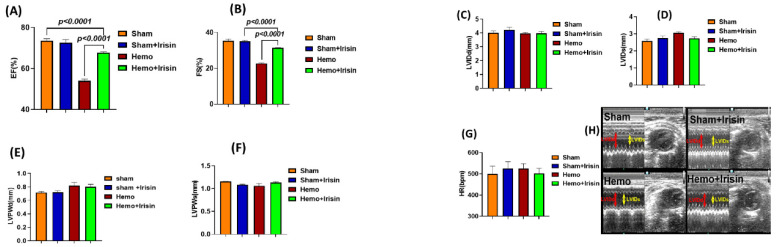
Echocardiographic measurements of cardiac function in hemorrhagic mice; Left ventricular (LV) function was assessed by two-dimensional M-mode echocardiography in sham and hemorrhage groups 2 h after sham operation and resuscitation. Bar graphs for (**A**) Ejection Fraction (EF); (**B**) Fractional Shortening (FS); (**C**) LV Internal Dimension in diastole (LVIDd); (**D**) LV Internal Dimension in systole LVIDs; (**E**) Left Ventricular Posterior Wall in diastole (LVPWd); (**F**) LV Posterior Wall in systole (LVPWs); and (**G**) Heart Rate (HR); (**H**) A representative image for the two dimensional and M-mode (Data are shown as Mean ± SEM, n = 4/each group).

Hemorrhage and resuscitation were performed, as shown in Figure 1A. As shown in Figure 1B,C, there is no difference in the mean arterial pressure among the groups prior to hemorrhage. The resuscitation showed a trend to increase the recovery of mean arterial pressure, but the difference among groups did not reach any significance. Myocardial performance was assessed by two-dimensional, M-mode echocardiography in sham and hemorrhage groups, both before and two hours after the sham operation and resuscitation. The cardiac function did not show a difference in the baseline before the hemorrhage. Figure 2 shows the representative echocardiograms in the sham operation and resuscitation groups. As shown in Figure 2A,B, ejection fraction (EF) and fractional shortening (FS) were significantly suppressed in the hemorrhagic shock groups compared to the sham groups, but irisin treatment significantly restored EF and FS in the hemorrhage group compared to the vehicle treatment. However, there were no significant differences in ventricular dimensions and wall thickness, including V internal dimensions (LVID), posterior wall thicknesses (PW) diastole and systole (LVIDd, LVIDs, LVPWd, LVPWs), and heart rate (HR) between the hemorrhage and sham groups (Figure 2C–H).

### 2.2. Irisin Attenuates Insulin Resistance in Hemorrhage

**Figure 3:**
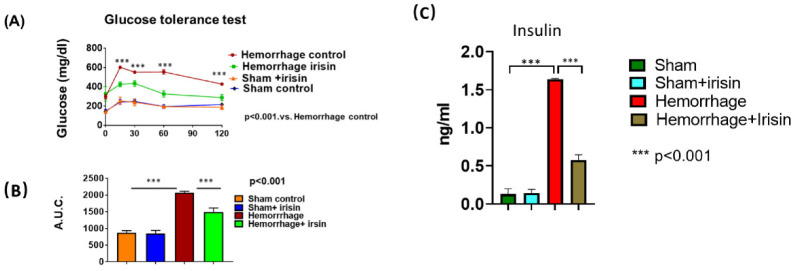
Glucose tolerance test and insulin measurement. Mice fasted 6 h before glucose tolerance test. (**A**) Time dependent glycemic levels post glucose injection (1.5 g/kg body weight of glucose was administered intraperitoneally. Data are shown as Mean ± SEM, n = 5/each group, *** *p* < 0.001); (**B**) A bar graph for blood glucose area under the curve analysis from GTT (Data are shown as Mean ± SEM, n = 5/each group, *** *p* < 0.001); (**C**) The measurement of insulin level in serum among the groups (Data are shown as Mean ± SEM, n = 5/each group, *** *p* < 0.001).

The glucose tolerance test was employed to examine insulin sensitivity in mice exposed to hemorrhage. Basal blood glucose levels were elevated in the hemorrhage group, and the delayed recovery of blood glucose levels after glucose loading was evident in hemorrhagic mice in the glucose tolerance test (GTT), which indicated the induction of insulin resistance in mice in response to the hemorrhage challenge. However, irisin treatment significantly attenuated glucose intolerance in the hemorrhage group (*p* < 0.001, Figure 3A), which is in line with an area under the curve (AUC) analysis of the GTT which reproduced the above results (*p* < 0.001, Figure 3B). In addition, the insulin level in the sera of hemorrhage was increased compared to that of the control, but irisin treatment attenuated the magnitude of insulin levels induced by the hemorrhage (Figure 3C). These results suggested that the hemorrhage elicited insulin resistance while irisin treatment protected the mice against hemorrhage-induced insulin resistance.

### 2.3. Irisin Attenuated Levels of Inflammatory Cytokines, IL-1 and TNF-α in Hemorrhage

**Figure 4:**
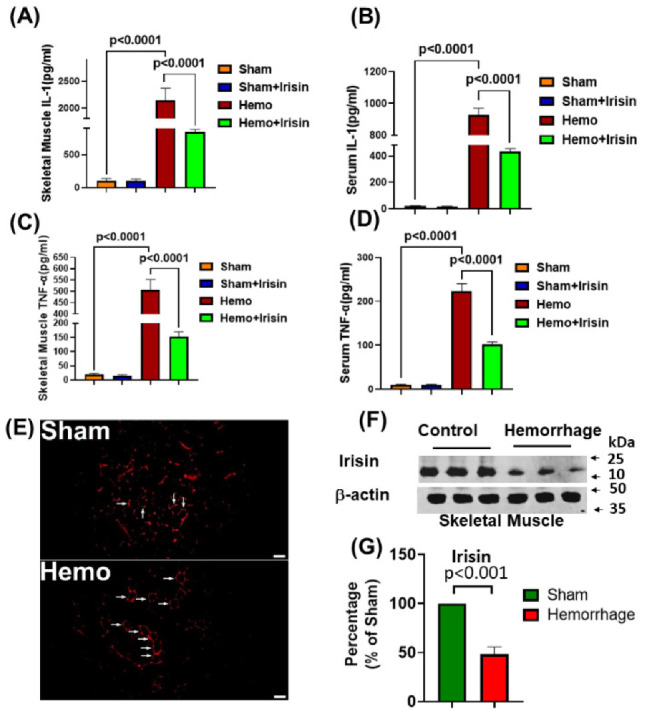
ELISA analysis of IL-1 and TNF-α in serum and skeletal muscle. Bar graphs for (**A**) Levels of IL-1 in skeletal muscle; (**B**) Serum levels of IL-1; (**C**) Levels of TNF-α in skeletal muscle; (**D**) Serum levels of TNF-α. (data are shown as Mean ± SEM, n = 4/each group); (**E**) Immunostaining showing that irisin signals were attenuated by hemorrhage, (Hemo: hemorrhage, Scale bar: 50µm); (**F**) Immunoblot of irisin and β actin in skeletal muscle of mice exposed to hemorrhage; (**G**) Densitometric analysis of irisin in hemorrhage and sham groups. Results were expressed as the percentage of sham group (Data are shown as Mean ± SEM, n = 5/each group).

It is known that hemorrhage triggers an inflammatory response that exacerbates pathological disorders. To assess the effect of hemorrhagic shock on the inflammatory response, Interleukin-1 (IL-1) and tumor necrosis factor-α (TNF-α) levels in serum and skeletal muscle lysate were measured in both sham and homogenized mice. As can be seen in Figure 4A–D, following two hours of resuscitation, the serum and muscle levels of TNF-α and IL-1 increased markedly in the hemorrhagic group, but the elevations of cytokine levels were reversed with irisin treatment, suggesting that irisin treatment attenuates the inflammatory response in IL-1 and TNF-α when induced by hemorrhage. In addition, we assessed the irisin contents produced in muscle: histochemical staining indicated that hemorrhage-suppressed irisin signals in the skeletal muscle tissue and western blot showed that, compared to sham controls, irisin proteins in the skeletal muscle were significantly reduced following the hemorrhage (Figure 4E–G).

### 2.4. Irisin Alleviated Histological Damage in Skeletal Muscle, and Cardiac Muscle and Lung in Hemorrhagic Shock

**Figure 5:**
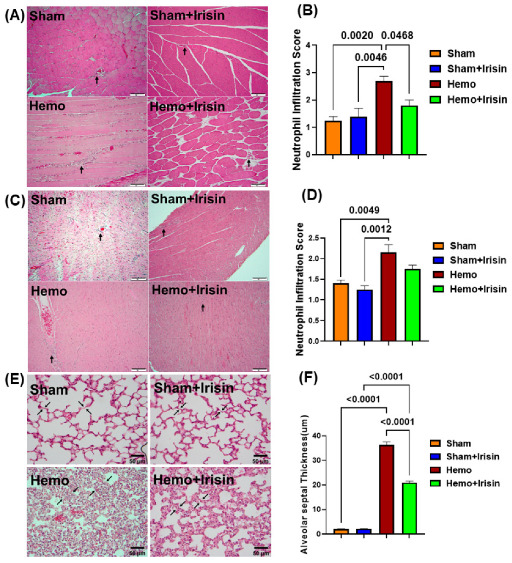
Histological analysis of injury in skeletal muscle, myocardium, and lung. (**A**) Skeletal muscle histology (H&E staining) of sham operated and hemorrhagic groups (200×, scale bars indicate 100 μm); (**B**) A bar graph for data of inflammatory cell infiltration in skeletal muscle (Data are shown as Mean ± SEM, n = 4/each group); (**C**) Cardiomyocyte histology (H&E staining) of sham operated and hemorrhagic groups (200×, scale bars indicate 100 μm); (**D**) A bar graph for data of neutrophilic infiltration in cardiac muscle (Data are shown as Mean ± SEM, n = 4/each group); (**E**) Histopathological images by H&E staining showed pathologic tissue damage in lungs from sham and hemorrhagic groups (H&E staining, original magnification, 400×, scale bars indicate 50 μm). Alveolar septal thickness was used to assess the extent of lung injury; (**F**) A bar graph for data of alveolar septal thickness of the lung (Data are shown as Mean ± SEM, n = 4/each group). Hemo: Hemorrhage.

Since hemorrhages are known to induce muscle edema, inflammatory cell infiltration, and vascular structure disruption, we examined the pathological changes in skeletal muscle, cardiac muscle, and the lungs by performing H&E staining of the tissues. Furthermore, as shown in Figure 5A–D, hemorrhage resulted in a more pronounced inflammatory cell infiltrate in cardiac and skeletal muscles, = compared to that in the sham group, and irisin treatment significantly alleviated inflammatory cell infiltration (*p* < 0.05). As shown in Figure 5E, significant thickening of the alveolar septal wall was observed in the hemorrhage group compared to the sham group, and irisin significantly reduced the degree of alveolar wall thickening (*p* < 0.0001) in the lungs (Figure 5F).

### 2.5. Irisin Reduced Apoptosis in Hemorrhage

**Figure 6:**
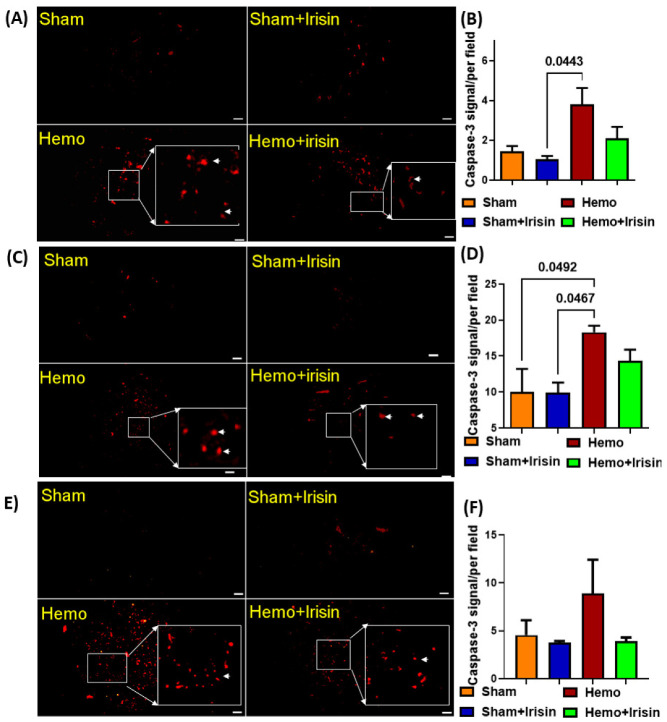
Immunostaining of active-caspase 3 signals in the skeletal muscle and cardiac muscle. (**A**) Immunohistochemical staining of caspase-3 in skeletal muscle (original magnification, 400×); (**B**) A bar graph for statistical data of positive staining of capase-3 from A (Data are shown as Mean ± SEM, n = 4/each group); (**C**) Immunohistochemical staining of caspase-3 in cardiac muscle (original magnification, 400×); (**D**) A bar graph for statistical data of positive staining of capase-3 from C; (**E**) Immunohistochemical staining of caspase-3 in lungs (original magnification, 400×); (**F**) A bar graph for statistical data of positive staining of capase-3 from (**E**) (Data are shown as Mean ± SEM, n = 4/each group). Hemo: Hemorrhage, Scale bar = 50 µm.

**Figure 7:**
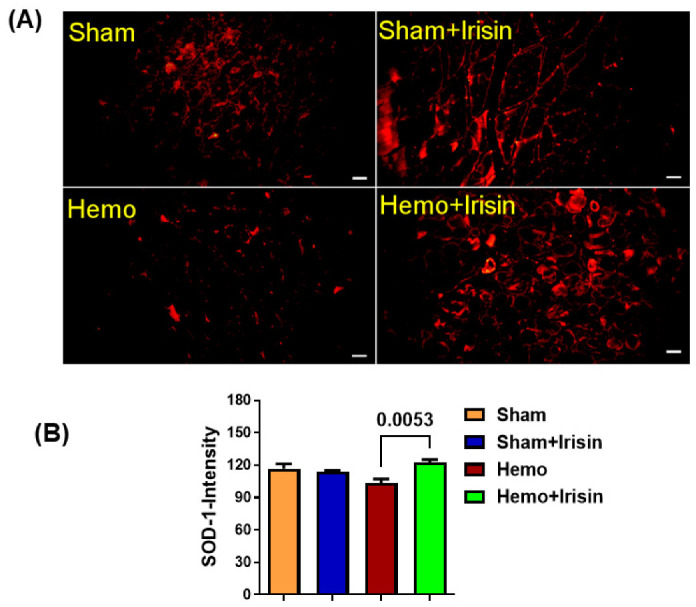
(**A**) Immunohistochemical staining of superoxide dismutase (SOD) in skeletal muscles; (**B**) A bar graph for statistical data of SOD intensity from A (data are shown as Mean ± SEM, n = 4/each group). Hemo: Hemorrhage, Scale bar: 50 µm.

Hemorrhages are considered to induce apoptosis in tissues. We employed immunofluorescent staining to examine the signal of active caspase 3 proteins in both skeletal and cardiac muscles. As shown in Figure 6, the hemorrhage group expressed higher levels of caspace-3 in skeletal muscles (Figure 6A,B) and cardiac muscles (Figure 6C,D) compared to that in the sham group. However, irisin treatment alleviated the elevated caspase-3 signals in the hemorrhage group, which did not reach a significant difference. Likewise, irisin also slightly suppressed caspase-3 positive signals in the lung tissue (Figure 6E,F), indicating that the hemorrhage induced an increase in active-caspase 3 signals, which could be suppressed by irisin treatment. In addition, there was a trend toward a decreased superoxide dismutase (SOD) in skeletal muscles (Figure 7A,B) in the hemorrhage group. Notably, SOD expression was increased in skeletal muscle when the hemorrhage group was treated with irisin. However, irisin treatment only resulted in a mild increase in SOD signaling in the cardiac muscles of the irisin + hemorrhage group compared to the hemorrhage alone (data not shown), suggesting that the increased SOD may be responsible for the protective effect of irisin.

## 3. Discussion

Hemorrhagic shock is a serious condition that can lead to multi-organ failure and high mortality in patients [13]. There is still much that remains to be clarified in the underlying mechanism of the disastrous course of events in hemorrhagic shock, because the pathophysiology which exacerbates the adverse outcomes is complex and involves multiple factors. Our salient study demonstrates that irisin plays a critical role in mitigating detrimental effects induced by hemorrhagic shock by improving myocardial performance, increasing insulin sensitivity, attenuating the inflammatory response, and reducing tissue damage in lungs and muscles.

It has been shown that hemorrhages result in metabolic disorders and insulin resistance, which contribute to the sequala of symptoms that result following a hemorrhage. The suppression of insulin resistance has demonstrated a protective effect against hemorrhage [14,15]. Our study revealed that mice, when exposed to hemorrhage, experienced glucose intolerance along with hyperinsulinemia, but the administration of irisin improved and attenuated the glucose intolerance induced by the hemorrhage and magnitude of hyperinsulinemia, indicating that irisin can ameliorate insulin sensitivity following a hemorrhage. Our previous studies, and others, have demonstrated that irisin-attenuated metabolic disorders, improved insulin sensitivity, and activated insulin signaling pathways contribute to the beneficial effects of irisin [15,16,17]. Although the precise molecular mechanism by which irisin mediates insulin sensitivity in hemorrhages is currently unknown, the improved insulin sensitivity by irisin plays a key role in protecting against hemorrhage.

Hemorrhages also lead to an overactivated immune system that releases pro-inflammatory cytokines [18,19,20]. In our study we confirmed this understanding and found that hemorrhages induced the expression of pro-inflammatory cytokines, TNF-α, and IL-1. TNF-α plays an important role in the inflammatory response under physiological stress with hemorrhagic shock [21]. In an inflammatory environment, TNF-α is produced primarily by locally infiltrated macrophages and monocytes. TNF-α has diverse biological activities, including the induction of apoptosis and promotion of neutrophilic migration into tissues [22,23]. Our results showed that neutrophil infiltration, as a marker of enhanced inflammatory response and tissue damage, was increased in the myocardial tissue and skeletal muscle after hemorrhagic shock. Likewise, our study also demonstrated elevated IL-1 levels in the sera and skeletal muscle tissue following hemorrhagic shock. IL-1 is a major inflammatory cytokine that plays a significant role in the regulation of inflammation during an immune response [24]. In our study, an acute administration of irisin in the resuscitation period reduced TNF-α and IL-1 levels in both serum and skeletal muscle tissue, suggesting the important contribution of irisin in suppressing inflammatory response and tissue injury in hemorrhage. The mechanisms by which irisin reduces inflammation in the hemorrhage remain unclear. It has been reported that irisin reduces the expression and activity of TNF-α and IL-6 in the pro-inflammatory activation of adipocyte 3T3 L1 cell lines and decreases the phosphorylation and activation of inflammatory transcription factors [25]. In addition, irisin alleviates pulmonary damage and decreases pulmonary microvascular permeability, which is related to the up-regulation of AMP-activated protein kinase and sirtuin 1 [26]. In agreement with this observation, our results demonstrated that hemorrhagic shock-induced alveolar septal thickening might contribute to acute lung injury, and that irisin treatment attenuated alveolar septal thickening, which is likely due to suppressed inflammatory response and apoptotic activities.

It is known that hemorrhages result in the depression of cardiac functions, which is considered to be one of the major factors that contribute to high mortality in patients [13]. Under the conditions of traumatic hemorrhage, a decline in cardiac performance is mirrored by the release of myocardial troponin I and biomarkers of myocardial injury [27,28]. In our studies, we have found that hemorrhages induce a depression in cardiac performance even after resuscitation. Irisin treatment resulted in an improvement in ejection fraction and fractional shortening. This is in agreement with our previous observation in which irisin protected the heart against ischemia and reperfusion injury [11], which is related to the irisin-induced up-regulation of myocardial SOD-1 and active-caspase 3. In addition, since TNF-α is one of the major factors that contribute to myocardial depression in the myocardial ischemia and infarction model, anti-treatment against TNF-α leads to an improvement in the function and attenuation of remodeling. In agreement with these previous observations, our studies indicate a correlation between cardiac depression and the increase of TNF-α and IL-1 in eliciting cardiac depression. Irisin-induced improvements to cardiac functions in this study were closely linked to the augmentation of cardiac functions, suggesting that attenuation in the production of TNF-α and IL-1 act as a mechanism for irisin’s cardiac effect in hemorrhage. In support of this observation, the irisin treatment of C2C12 skeletal myoblasts in our study enhanced C2C12 myotube differentiations and attenuated the cytotoxicity of muscles exposed to LPS (inflammatory stress), suggesting that irisin expresses anti-inflammatory effects which may contribute to improved cardiac functions (data not shown). Irisin was previously found to improve myocardial functions in the diabetic heart in association with an improvement in insulin sensitivity and the PRAK pathway [29], revealing that irisin-enhanced insulin sensitivity modulates cardiac performance in metabolic disorders. Irisin-induced improvements to cardiac functions following a hemorrhage here are associated with an increase in insulin sensitivity, suggesting that insulin sensitivity could be an additional factor in the regulation of cardiac performance.

The massive blood loss seen in hemorrhagic shock induces apoptosis via an increase in reactive oxygen species (ROS) generation in several models [30,31,32,33,34]. Inhibiting ROS generation [35,36] or enhancing the superoxide dismutase type 1 (SOD-1), a free radical scavenging enzyme, becomes implicated in ischemia/reperfusion injury [36]. Irisin elevated the SOD expression in response to oxidative stress [37] and ameliorated ROS-induced endothelial dysfunctions in obesity [38]. In this observation, SOD-1 levels in muscle tissue were slightly suppressed in the hemorrhagic shock group, which were prevented by irisin treatment, suggesting that an irisin-induced increase in SOD may be associated with its protective effects against hemorrhages. The induction of intrinsic apoptotic signaling factors through caspase-3 by hemorrhagic shock has also been reported [30,39]. In our study, active-caspase 3 positive signaling in skeletal and cardiac muscle tissues was increased in the hemorrhage group, which was slightly suppressed following irisin treatment, suggesting that irisin may have anti-apoptotic effects in hemorrhages.

## 4. Materials and Methods

### 4.1. Animals

Two-month-old male CD-1 mice were purchased and used for all experiments (The Charles River Laboratory, ME). These animals were housed in an accredited facility at Rhode Island hospital (Providence, RI, USA). All studies on animals were conducted under a protocol approved by the Institutional Animal Care and Use Committee of Rohde Island hospital, which conforms to the Guide for the Care and Use of Laboratory Animals published by the US National Institutes of Health (NIH Publication No. 85–23, revised 1996).

Animal ventilation and surgical procedure: anesthesia was induced with 1–4% isoflurane in oxygen. Mice were then transferred to the nose cone. Once non-responsive to toe-pinch, surgical prep was performed in the left and right inguinal areas. A rectal thermometer was inserted to evaluate body temperature, and the animal was maintained on a warming pad to maintain body temperature. We monitored anesthesia at a minimum of every 10–15 min during the procedure. Once the prep was complete, diluted lidocaine was injected (not to exceed 5 mg/kg total dose) under the skin of the anticipated incision. Using sharp scissors, starting immediately in the medial area of the thigh, a 2 cm long straight incision was made from the knee towards the medial thigh to fully expose the area overlying the femoral artery. The tissues were bluntly dissected by gently separating the skin from the underlying tissue circumferentially around the entire incision site. The area was kept moist with warm sterile saline. The underlying tissue was gently separated using blunt dissection to expose the muscular layer for visualization of the femoral artery. After dissection, a 7-0 silk suture was passed underneath the distal end of the femoral artery towards the knee using double knots, which were used to create tension in order to achieve full arterial occlusion. Another larger length 7-0 silk suture was placed in a more rostral position, leaving enough length for a second tie to be used to eventually secure the pressure catheter (PE-10 tube) in place. At the same time, microvascular clamps were placed underneath the artery to aid with pressure catheter positioning for pressure measurement. A similar procedure without using the pressure transducer was performed on the contralateral leg in order to facilitate cannulation, to be used for the hemorrhage procedure. Hemorrhage was then induced by removing blood from the right femoral artery until mean arterial blood pressure (MABP) reached 40 mmHg. The left femoral artery was used for continuous MABP monitoring. If the MABP surpassed 45 mmHg, additional blood was withdrawn from the right femoral artery. If the MABP dropped below 35 mmHg, an infusion of 0.5 mL of lactated ringers solution was infused into the right femoral artery. Withdrawn blood was collected into sterile tubes with citrate at a ratio of 0.1 mL citrate/1 mL sterile blood to prevent clotting. This blood would be used later for infusion via the right femoral artery after the procedure was finished to replace blood volume via the same catheter. The mice were kept at a MABP range of between 35 mmHg and 45 mmHg for 60 min.

### 4.2. Experimental Group Design

At the end of the 60 min of hemorrhage, mice were resuscitated with their shed blood and three times the volume of total blood loss with LRS or saline, using a sterilized syringe over a 10-minute period. The resuscitation fluids were injected through the femoral catheter as described in the above procedure. The mice were then assigned to either the *irisin treatment group*, which would receive 50 ng/kg of intravenous irisin, or to a *vehicle control*. After the treatment, the catheters were removed, and the incisions were sutured to close. *Sham-operated* mice were anesthetized and underwent cannulation but without blood withdrawal. A single 0.5 mg/kg body weight dose of subcutaneous buprenorphine was given for analgesia for all mice in the study. Two hours after resuscitation, echocardiography was carried out to measure cardiac function under anesthesia, induced with 1–4% of isoflurane in oxygen as above. The mice were then euthanized, and blood and tissue were collected for immunological and biochemical analysis. A total of 25 mice were used in this study, of which five mice were excluded because of catheterization failure. The diagram of the experiment is shown in Figure 1A.

### 4.3. Echocardiographic Measurements

The echocardiography of mice was performed using the CX50 system equipped with a L15-7io broadband linear array (Philips, Cambridge, MA, USA) to access cardiac function. Mice were anesthetized with continuous inhalation of 1–4% isoflurane via a nose cone. Mice were placed in the supine position on a heating pad. Hair was removed by a trimmer and Nair hair remover. A pre-warmed ultrasound transmission gel (Aquasonic, Parker Laboratory, Fairfield, NJ, USA) was then applied to the precordial region. The short axis of the left ventricle was captured at the level of the papillary muscles for two-dimensional B-mode and M-mode images. Between three and six consecutive cardiac cycles were used to assess the dimension and heart rate through the M-mode. The measurements were performed by an experienced operator in a double-blinded manner. At the end of echocardiography, the mice were euthanized using 5% isoflurane which was confirmed by bilateral thoracotomy.

### 4.4. Glucose Tolerance Test (GTT)

The measurement of glucose tolerance was performed to determine insulin resistance. The mice fasted for six hours, and tail vein blood was collected to measure the basal glucose level using an Accu-Chek Compact Plus glucometer (Roche Diagnostics, Indianapolis, IN, USA). The mice then received a subsequent injection of glucose at a dose of 1.5 mg/kg intraperitoneally. Glucose levels in the tail vein blood were measured at 15, 30, 60, and 120 min after the glucose injection.

### 4.5. Measurement of Cytokines Using Enzyme-Linked Immunoassay (ELISA)

IL-1 and TNF-α levels in sera and tissue lysates were measured using a commercially available enzyme-linked immunosorbent assay (ELISA) kit (R & D systems, Minneapolis, MN, USA). Assays were performed following the manufacturer’s instructions. Briefly, a 10× diluted serum or 1.5 mg/mL tissue protein along with known concentrations of cytokine standards was applied to the primary antibody pre-coated 96-well ELISA plates. After overnight incubation at 4 °C, a biotinylated secondary antibody was applied and incubated for oneβ hour at room temperature. After the incubation, streptavidin-horseradish peroxidase (SA-HRP) solution was applied, followed by tetramethylbenzidine (TMB) substrate solution. Concentrations of the cytokines were estimated from the absorbance at 450 nm using a spectrophotometer (SpectraMax M2e Multi-Mode Microplate Reader, Molecular Device, LLC, San Jose, CA, USA). Protein concentrations in the tissue lysates were quantified using the bicinchoninic acid protein assay kit (Thermo Fisher Scientific, Waltham, MA, USA).

### 4.6. Measurement of Blood Insulin Content Using Enzyme-Linked Immunoassay (ELISA)

Insulin content in the blood was also measured using a Mouse Insulin ELISA kit from ALPCO (Salem, NH, USA) according to the instructions of the manufacturer. The level of insulin was determined using a standard curve obtained from standards provided by the kit.

### 4.7. Western Blotting

Protein levels from skeletal muscles were measured by western blotting, using tissue lysates (50 μg/lane) as described in our previous methods [40]. In brief, the blots were incubated with their respective polyclonal antibodies, which included polyclonal rabbit irisin (1:1000, Abcam, #13847, Waltham, MA, USA) and polyclonal rabbit beta-actin at a diluted concentration of 1:1000. Both antibodies have been calibrated to work well in our previous work. The signals were then visualized by an anti-rabbit horseradish peroxidase-conjugated secondary antibody (1:2000). The results were visualized with a Super Signal West Pico ECL chemiluminescence reagent (Thermo-Fisher Scientific, Waltham, MA, USA). The immunoblotting signals were visualized by a Super Signal West Pico ECL chemiluminescence reagent (Thermo-Fisher Scientific, Waltham, MA, USA). The irisin levels were detected against a polyclonal rabbit antibody (Abcam). Densitometric analysis for the blots was conducted using NIH Image J software version 1. 5. 3.

### 4.8. Histological and Immunochemical Analysis

Freshly isolated lung, skeletal muscle, and heart tissue were placed in 10% formalin for 24 to 48 h to fix prior to processing. After fixation, the tissues were dehydrated with gradient alcohol and then embedded in paraffin for thin sectioning. Hematoxylin and eosin (H&E) staining was used for the evaluation of the alveolar septal thickness in the lungs and neutrophil infiltrations in the skeletal muscle and the heart tissue samples. Results were scored by two independent pathologists. For immunostaining, paraffin-embedded sections were dewaxed and hydrated for 10 min at room temperature. They were then incubated with the rabbit polyclonal irisin antibody (1:200, Abcam, #13847, Waltham, MA, USA), rabbit polyclonal anti-caspase-3, and rabbit polyclonal anti-SOD-1 primary antibodies overnight at 4 °C. Sections were then washed 3 times in phosphate buffer saline (PBS) before being incubated with the secondary antibodies (CyTM3-conjugated goat anti-rabbit IgG, 1:200, Thermo Fisher Scientific, Burlington, MA, USA) for one hour in the dark. After washing with PBS, a mouse monoclonal anti-MF-20 antibody (1:200, Thermo Fisher Scientific #14-6503-82) was added and incubated for two hours at room temperature. After sections were washed three times with PBS, an FITC-conjugated goat anti-mouse IgG secondary antibody (1:200, Thermo Fisher Scientific, F-2761) was added and incubated for an additional one hour in the dark, at room temperature, followed by three additional washes with PBS. After the final wash, 4′,6-diamidino-2-phenylindole (DAPI; Thermo Fisher Scientific) was applied for nuclear staining, and samples were washed three times. Slides were then mounted with DABCO (Millipore-Sigma) and sealed with oil. The immunofluorescent images were captured using a laser scanning microscope. Approximately five—ten randomly selected fields in each section were used for quantification in H&E staining and immunohistochemical analysis using NIH image J software.

### 4.9. Statistical Analysis

The data are presented as means ± SE. Data were analyzed by one-way ANOVA, with the post hoc Tukey test used for pairwise comparisons. Statistical analysis was performed by Graphpad Prism 9 software (GraphPad Software, San Diego, CA, USA). A *p*-value < 0.05 was considered significant.

## 5. Conclusions

We have demonstrated that irisin plays a critical role against hemorrhage-induced detrimental effects, as indicated by an improvement in myocardial performance and the promotion of insulin tolerance. Hemorrhage-elicited insulin resistance was attenuated by irisin treatment, which contributed to functional improvement. Furthermore, the hemorrhage induced the release of muscle and peripheral cytokines, which were suppressed by irisin; this suggests that anti-inflammation by irisin serves as a key regulator for its protective roles. Our study provides direct evidence showing that irisin functions as a critical regulator for protecting against hemorrhages and holds promise in developing a novel therapeutic treatment for multiple organ dysfunction.

## Data Availability

Data is contained within the article.

## References

[1] Cannon J.W. (2018). Hemorrhagic Shock. N. Engl. J. Med..

[2] Heckbert S.R., Vedder N.B., Hoffman W., Winn R.K., Hudson L.D., Jurkovich J.L., Copass M.K., Harlan J.M., Rice C.L., Maier R.V. (1998). Outcome after hemorrhagic shock in trauma patients. J. Trauma.

[3] Bostrom P., Wu J., Jedrychowski M.P., Korde A., Ye L., Lo J.C., Rasbach K.A., Boström E.A., Choi J.H., Long J.Z. (2012). A PGC1-alpha-dependent myokine that drives brown-fat-like development of white fat and thermogenesis. Nature.

[4] Maak S., Norheim F., Drevon C.A., Erickson H.P. (2021). Progress and Challenges in the Biology of FNDC5 and Irisin. Endocr. Rev..

[5] Ye X., Shen Y., Ni C., Ye J., Xin Y., Zhang W., Ren Y.Z. (2019). Irisin reverses insulin resistance in C2C12 cells via the p38-MAPK-PGC-1α pathway. Peptides.

[6] Zheng S., Chen N., Kang X., Hu Y., Shi S. (2022). Irisin alleviates FFA induced β-cell insulin resistance and inflammatory response through activating PI3K/AKT/FOXO1 signaling pathway. Endocrine.

[7] Li D.J., Sun S.J., Fu J.T., Ouyang S.-X., Zhao Q.-J., Su L., Ji Q.-X., Sun D.-Y., Zhu J.-H., Zhang G.-Y. (2021). NAD(+)-boosting therapy alleviates nonalcoholic fatty liver disease via stimulating a novel exerkine Fndc5/irisin. Theranostics.

[8] Zhu W., Sahar N.E., Javaid H.M.A., Pak E.S., Liang G., Wang Y., Ha H., Huh J.Y. (2021). Exercise-Induced Irisin Decreases Inflammation and Improves NAFLD by Competitive Binding with MD2. Cells.

[9] Wang J., Zhao Y.T., Zhang L., Dubielecka P.M., Zhuang S., Qin G., Chin Y.E., Zhang S., Zhao T.C. (2020). Irisin Improves Myocardial Performance and Attenuates Insulin Resistance in Spontaneous Mutation (Lepr (db)) Mice. Front. Pharmacol..

[10] Xu J., Kim H.T., Ma Y., Zhao L., Zhai L., Kokorina N., Wang P., Messina J.L. (2008). Trauma and hemorrhage-induced acute hepatic insulin resistance: Dominant role of tumor necrosis factor-alpha. Endocrinology.

[11] Wang H., Zhao Y.T., Zhang S., Dubielecka P.M., Du J., Yano N., Chin Y.E., Zhuang S., Qin G., Zhao T.C. (2017). Irisin plays a pivotal role to protect the heart against ischemia and reperfusion injury. J. Cell Physiol..

[12] Zhao Y.T., Wang J., Yano N., Zhang L.X., Wang H., Zhang S., Qin G., Dubielecka P.M., Zhuang S., Liu P.L. (2019). Irisin promotes cardiac progenitor cell-induced myocardial repair and functional improvement in infarcted heart. J. Cell Physiol..

[13] Gutierrez G., Reines H.D., Wulf-Gutierrez M.E. (2004). Clinical review: Hemorrhagic shock. Crit Care.

[14] Raje V., Ahern K.W., Martinez B.A., Howell N.L., Oenarto V., Granade M.E., Kim J.W., Tundup S., Bottermann K., Gödecke A. (2020). Adipocyte lipolysis drives acute stress-induced insulin resistance. Sci. Rep..

[15] Jiang S., Gavrikova T.A., Messina J.L. (2014). Regulation of hepatic insulin receptor activity following injury. Am. J. Physiol. Gastrointest. Liver Physiol..

[16] Zhang M., Xu Y., Jiang L. (2019). Irisin attenuates oxidized low-density lipoprotein impaired angiogenesis through AKT/mTOR/S6K1/Nrf2 pathway. J. Cell Physiol..

[17] Medhat D., El-Bana M.A., El-Daly S.M., Ashour M.N., Elias T.R., Mohamed R.A., Yassen N.N., Abdel-Monem M.A., Hussein J. (2021). Influence of irisin on diet-induced metabolic syndrome in experimental rat model. J. Complement Integr. Med..

[18] Albertsmeier M., Pratschke S., Chaudry I., Angele M.K. (2014). Gender-Specific Effects on Immune Response and Cardiac Function after Trauma Hemorrhage and Sepsis. Viszeralmedizin.

[19] Chu X., Schwartz R., Diamond M.P., Raju R.P. (2019). A Combination Treatment Strategy for Hemorrhagic Shock in a Rat Model Modulates Autophagy. Front. Med..

[20] Raju R. (2017). Immune and metabolic alterations following trauma and sepsis—An overview. Biochim. Biophys. Acta Mol. Basis Dis..

[21] Vallejo J.G., Nemoto S., Ishiyama M., Yu B., Knuefermann P., Diwan A., Baker J.S., Defreitas G., Tweardy D.J., Mann D.L. (2005). Functional significance of inflammatory mediators in a murine model of resuscitated hemorrhagic shock. Am. J. Physiol. Heart Circ. Physiol..

[22] Boyle J.J., Weissberg P.L., Bennett M.R. (2003). Tumor necrosis factor-alpha promotes macrophage-induced vascular smooth muscle cell apoptosis by direct and autocrine mechanisms. Arter. Thromb. Vasc. Biol..

[23] Parameswaran N., Patial S. (2010). Tumor necrosis factor-alpha signaling in macrophages. Crit. Rev. Eukaryot. Gene Expr..

[24] Kaneko N., Kurata M., Yamamoto T., Morikawa S., Masumoto J. (2019). The role of interleukin-1 in general pathology. Inflamm. Regen..

[25] Mazur-Bialy A.I. (2019). Superiority of the Non-Glycosylated Form Over the Glycosylated Form of Irisin in the Attenuation of Adipocytic Meta-Inflammation: A Potential Factor in the Fight Against Insulin Resistance. Biomolecules.

[26] Li X., Jamal M., Guo P., Jin Z., Zheng F., Song X., Zhan J., Wu H. (2019). Irisin alleviates pulmonary epithelial barrier dysfunction in sepsis-induced acute lung injury via activation of AMPK/SIRT1 pathways. Biomed. Pharmacother..

[27] Wall J., Naganathar S., Praditsuktavorn B., Bugg O.F., McArthur S., Thiemermann C., Tremoleda J.L., Brohi K. (2019). Modeling Cardiac Dysfunction Following Traumatic Hemorrhage Injury: Impact on Myocardial Integrity. Front. Immunol..

[28] De’Ath H.D., Rourke C., Davenport R., Manson J., Renfrew I., Uppal R., Davies L.C., Brohi K. (2012). Clinical and biomarker profile of trauma-induced secondary cardiac injury. Br. J. Surg..

[29] Du J., Zhao Y.T., Wang H., Zhang L.X., Qin G., Zhuang S., Kadin M., Chin Y.E., Liu P.Y., Zhao T.C. (2021). The Essential Role of PRAK in Preserving Cardiac Function and Insulin Resistance in High-Fat Diet-Induced Diabetes. Int. J. Mol. Sci..

[30] Childs E.W., Tharakan B., Hunter F.A., Tinsley J.H., Cao X. (2007). Apoptotic signaling induces hyperpermeability following hemorrhagic shock. Am. J. Physiol. Heart Circ. Physiol..

[31] Fan J., Li Y., Levy R.M., Fan J.J., Hackam D.J., Vodovotz Y., Yang H., Tracey K.J., Billiar T.R., Wilson M.A. (2007). Hemorrhagic shock induces NAD(P)H oxidase activation in neutrophils: Role of HMGB1-TLR4 signaling. J. Immunol..

[32] Childs E.W., Udobi K.F., Wood J.G., Hunter F.A., Smalley D.M., Cheung L.Y. (2002). In vivo visualization of reactive oxidants and leukocyte-endothelial adherence following hemorrhagic shock. Shock.

[33] Zuo L., Zhou T., Pannell B.K., Ziegler A.C., Best T.M. (2015). Biological and physiological role of reactive oxygen species—The good, the bad and the ugly. Acta Physiol..

[34] Kalogeris T., Baines C.P., Krenz M., Korthuis R.J. (2016). Ischemia/Reperfusion. Compr. Physiol..

[35] Granger D.N., Kvietys P.R. (2015). Reperfusion injury and reactive oxygen species: The evolution of a concept. Redox Biol..

[36] Wu L., Xiong X., Wu X., Ye Y., Jian Z., Zhi Z., Gu L. (2020). Targeting Oxidative Stress and Inflammation to Prevent Ischemia-Reperfusion Injury. Front. Mol. Neurosci..

[37] Mazur-Bialy A.I., Pochec E. (2021). The Time-Course of Antioxidant Irisin Activity: Role of the Nrf2/HO-1/HMGB1 Axis. Antioxidants.

[38] Han F., Zhang S., Hou N., Wang D., Sun X. (2015). Irisin improves endothelial function in obese mice through the AMPK-eNOS pathway. Am. J. Physiol. Heart Circ. Physiol..

[39] McIlwain D.R., Berger T., Mak T.W. (2013). Caspase functions in cell death and disease. Cold Spring Harb. Perspect Biol..

[40] Yano N., Zhang L., Wei D., Dubielecka P.M., Wei L., Zhuang S., Zhu P., Qin G., Liu P.Y., Chin Y.E. (2020). Irisin counteracts high glucose and fatty acid-induced cytotoxicity by preserving the AMPK-insulin receptor signaling axis in C2C12 myoblasts. Am. J. Physiol. Endocrinol. Metab..

